# The Use of the Model of Occupational Self-Efficacy for Work Retraining: A Multiple Case Study

**DOI:** 10.1155/2019/3867816

**Published:** 2019-10-01

**Authors:** Mogammad Shaheed Soeker, Candice Pape

**Affiliations:** Department of Occupational Therapy, University of the Western Cape, Cape Town 7535, South Africa

## Abstract

The South African labour law serves as a guide for employers to accommodate injured individuals in the work place. The aim of the study was to explore and describe the experiences of individuals with traumatic brain injury regarding returning to work through the use of the Model of Occupational Self-Efficacy (MOOSE). The study utilized a multiple case study research design, and 10 participants participated in the study. An analytical strategy of explanation building was used to analyse the data. Three themes emerged from the study, i.e., Theme 1: the model has its limitations (barriers), Theme 2: the model helps facilitate work integration (facilitators), and Theme 3: further enhancements to improve the model. The findings of this study indicated that the participant experiences of the MOOSE are a useful model to facilitate the return of individuals living with a TBI back to work. Coping skills and support groups were also viewed as being an important part of the vocational rehabilitation program.

## 1. Introduction

A traumatic brain injury can cause an individual to experience long-term physical, cognitive, and psychosocial impairments [1]. For individuals who have mild or moderate levels of TBI, the symptoms may not be obvious to the casual observer, and therefore, it is often called the “hidden disability” [2], whereas individuals with severe injuries may experience more severe physical, cognitive, and psychological losses. According to the National Institute for Occupational Health [3], of a population of 49.3 million, 5% were recorded disabled. A study in 2007 found injury-related mortality rates in South Africa to be 6 times higher, and the incidence of road traffic injuries to be double, than that of the global rate [4]. The major risk factors for TBI are extremes of age, male gender, and low socioeconomic status. In the US, the leading causes of TBI are falls, followed by MVAs, being struck by/against objects, and assaults. In the medical model, the individual with an injury or disability is regarded as having problems that require mainly medical-biological and in condoning the relief of disability's burdens through medical rather than environmental or attitudinal interventions [5], One criticism of the medical model includes the fact that disability is considered a condition that needs to be normalised, often disregarding the personal autonomy of the person with disability to choose to live with the disability in society [5]. Another criticism relates to the medical model that it mainly assumes the patient or client to be a passive recipient of care, with minimal input in terms of planning intervention [6]. There is therefore a need for studies to explore and obtain the perspectives and experiences of the patient or client who is the main recipient of care. Most studies focus mainly on the outcomes of vocational rehabilitation programs such as return to work, and these outcomes are mainly measured with questionnaires. The current study seeks to obtain the perspectives of individuals about the usefulness of the Model of Occupational Self-Efficacy (MOOSE) after they participated in the stages of the MOOSE. The Model of Occupational Self-Efficacy is the first occupational therapy-based model that has specifically been developed for individuals who sustained a traumatic brain injury. The model differs from other occupational therapy models of practice as it focuses specifically on improving an individual's work-related skills, it encourages introspection and goal setting, and most importantly, it encompasses elements that is unique to supportive employment work-related models [7].

## 2. Literature Review

Holistically, there is evidence that traumatic brain injury negatively affects postinjury employment status. In a systematic review that focused on vocational rehabilitation for individuals with TBI conducted by Stergiou and Dawson [8], it was found that large numbers of individuals with TBI are unemployed and that the return to work rate for individuals with brain injury was only about 40%, one to two years post injury. Postinjury, victims of brain injury have to endure a variety of limitations, which limit their ability to adapt to their worker role. Wood and Worthington [9] indicated that individuals who sustained a traumatic brain injury (TBI) experience problems with executive functions that impair their ability to engage in daily life activities and engage in social activities and the individual's ability to return to work successfully. Due to the latter losses, they no longer see themselves as contributing members of society. It could therefore be argued that these individuals with brain injury are no longer able to fill their roles as breadwinners in their families.

**Figure 1:**
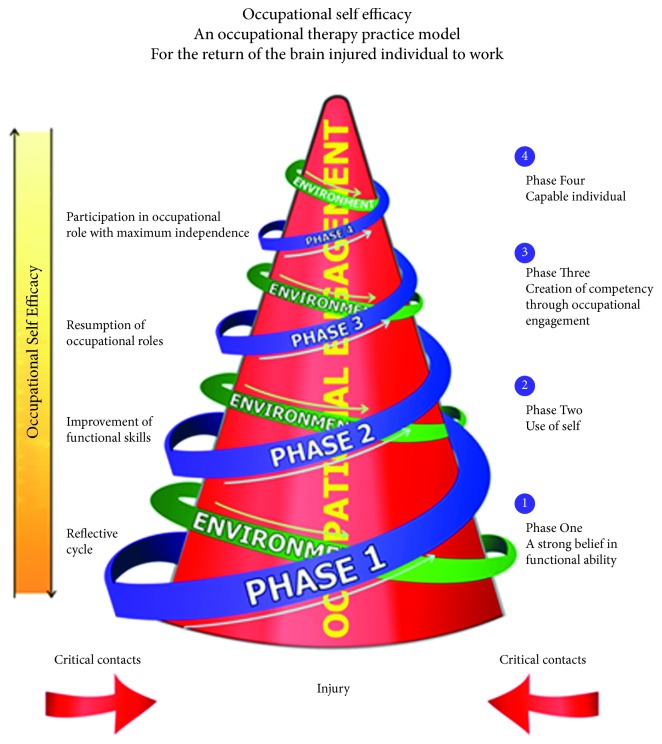
Model of Occupational Self-Efficacy by M.S. [11], *Work*, 53, p. 526. Copyright 2016 IOS Press. Reprinted with permission.

Occupational therapy is a profession that offers holistic care management and interventions for individuals that experience physical, psychological, emotional, and social limitations associated with disability and functioning in their daily occupations [10]. The Model of Occupational Self-Efficacy advanced by Soeker [11] is an occupational therapy practice model designed to effectively return individuals with brain injury to work. According to Soeker [11], the MOOSE consists of 4 stages (see Figure 1), namely, stage one: a strong personal belief in functional abilities—the occupational therapist will facilitate a process of enabling the individual with brain injury (or any disability) to reflect on their limitations and work abilities in order to cope in their work and social environment. Stage two: use of self—the occupational therapist focuses on improving the individual who sustained a brain injury's self-esteem and motivation to participate in work and everyday tasks. Stage three: creation of competency through occupational engagement—the occupational therapist would create an environment that will enable the individual with brain injury to enhance specifically his work-related skills, often simulating the work tasks and work environment that the individual participated in before their injury. Stage four: capable individual—the occupational therapist will enable the individual with brain injury to engage in work tasks in the open labour market more independently; usually, in this stage, the assistance and support are minimal from the occupational therapist, therefore allowing the individual with brain injury to problem solve the various challenges that he may experience in the work place. It could therefore be argued that the Model of Occupational Self-Efficacy could be unique when compared to other occupational therapy models in that it is client-centered, allows for reflection by the individual with injury, and focuses on building an individual's self-efficacy beliefs. The proposed research aims to explore the experiences of individuals with brain injury regarding the use of the model in enabling them to return to work after their participation in a vocational rehabilitation program using the MOOSE.

## 3. Aim

The aim of the study is to explore and describe the experiences of individuals with TBI regarding returning to work through the use of the Model of Occupational Self-Efficacy.

## 4. Objectives

The objectives of this study were as follows:

- To describe the barriers that individuals with TBI experience when returning to work after utilizing the Model of Occupational Self-Efficacy
- To describe the enablers that individuals with TBI experience when returning to work after utilizing the Model of Occupational Self-Efficacy

## 5. Research Design

The current study was positioned in the qualitative paradigm; a case study approach was utilized that was exploratory in nature. The current study utilized the multiple case study design as advocated by Yin [12]; it was used to explore the experiences of individuals with brain injury regarding the use of the Model of Occupational Self-Efficacy (MOOSE). The current study is unique as it is the first study of its kind that utilized Yin's case study design in the application of the Model of Occupational Self-Efficacy.

## 6. Population and Sampling

Ten participants were purposively sampled from the statistical records from the Occupational Therapy departments of Tertiary Hospitals and Community Health Centers. In order to contextualize the findings of the study, the demographics of the participants in this study will be presented in a table form (see Table 1).

### 6.1. Inclusion Criteria

The inclusion criteria were as follows. Participants were diagnosed with either a mild or a moderate brain injury, they must have been employed for remuneration before their injury and at least 3 months post rehabilitation, and they must be able to communicate effectively in English and Afrikaans and be able to understand verbal questions. They were also required to be over 18 years old.

### 6.2. Exclusion Criteria

Participants who had sustained severe head injuries were excluded as literature revealed that the probability of their reintegrating into the open labour market worker role would be implausible. Members who had active symptoms related to additional psychiatric disorders according to the DSM V and individuals with multiple disabilities were also excluded.

## 7. The Application of the MOOSE to Clients with Traumatic Brain Injury

The participants participated in all four stages of the MOOSE, for the purpose of improving their work skills [11]. Stage one and stage two were applied to the study in the following manner. The researcher focused on building interpersonal relationships, building a positive self-image, goal setting, problem solving, and activities to enhance their memory. In stage three, the participants participated in more work-related types of activities such as administration tasks, computer work, and packing activities. The participants were given the opportunity to practice their work skills in a real work setting under supervision (work test placement). In stage four, the participants were placed in the work setting for longer periods of time, i.e., up to 6 months often in paid employment. During stage four, the support that was given to the participants was gradually reduced until the participants were capable of working independently. Each phase of the program consisted of an average of six sessions that were one hour in duration. During these sessions, the participants would participate in individual and group sessions.

## 8. Data Analysis and Rigor

The researcher utilized the data analysis strategy for explanation building as advocated by Yin [12]. In view of this analysis, transcriptions of each interview were coded, categorized, and placed into themes in order to conceptualize the information gathered. During the coding process, the researcher utilized observation and field notes to ensure the validity of the study.

Strategies such as credibility, transferability, dependability, and confirmability were used in order to ensure the trustworthiness of the data [13]. Credibility was ensured by means of member checking and triangulation. Transferability was ensured by the detailed description of the research methods and contexts and detailed description of the participants and the experience of the participants. Dependability was ensured by means of dense descriptions, peer examination, and triangulation. Confirmability was ensured by the process of reflexivity whereby the researcher's own biases or assumptions were made apparent by means of a reflexive journal.

## 9. Data Collection

Data was collected by means of face to face, semistructured interviews, and simple observation methods. The duration of the interviews was between 45 and 60 minutes, and one interview was conducted at each phase of the model. As the model had 4 phases, 4 interviews were conducted with each research participant, using Yin's [12] 5 steps of research design. Spradley [14] describes *simple observation* as studying and observing people's behaviors and attitudes through the observation of certain simple tasks before more detailed complicated tasks. The simple observation method was implemented whereby the brain-injured individual was observed during the rehabilitative stages of the model (stages 1-2) as well as when they were employed in the latter stages of the model (stages 3-4). Documents that related to the participant medical information and history were obtained from various sources of knowledge (i.e., other health professionals and medical records from the hospital).

### 9.1. Ethics

The principles related to ethics as described in the Helsinki Declaration were utilized in the current study [15]. The researcher obtained ethics approval from the Research Ethics Committee of the University of the Western Cape, followed by permission from the Medical Superintendent at Tygerberg Hospital in order to conduct the study in the Occupational Therapy Department. Informed written consent from participants was obtained, and they were informed that confidentiality and their right to remain anonymous were ensured. Participants had the right to withdraw from the study at any time, and they were under no obligation to continue participating in the study. Furthermore, they were informed that should they require any assistance in the form of counselling or medical intervention then they would be referred to an appropriate source.

## 10. Findings

The current findings will be presented by means of three themes and its associated categories.

### 10.1. Theme 1: The Model Has Its Limitations (Barriers)

The theme above is representative of the participant's experiences in relation to the limitations of using the model.

#### 10.1.1. Category 1: “Stage Two Is Too Frustrating”

The category named “stage two is too frustrating” was derived from the general perception that participants found stage 2 to be very challenging and frustrating as they were improving their work abilities (memory, concentration, and appropriate social behavior). They displayed negative attitudes and a low volition towards paper-based activities, work sheets, and implementing memory techniques. One participant described the activities in stage 2 as

Yes we were learning, but we were doing small things. Those things we were doing here was so small… (Isaiah)

He further suggested that he would prefer manual labour and expressed the need to feel tired after “working” in order to derive satisfaction from the treatment session. He continued:

Yes, and maybe someone will ask me what I did today then I can tell them I was working hard today. (Isaiah)

#### 10.1.2. General Lack of Resources and Employment Opportunities: “What's the Point”

Participants expressed their desperation as none of them had an income, the program itself is a lengthy process, and most struggled to get transport fees in order to attend their sessions; in addition, they also often attended sessions hungry due to poor social economic conditions. This made it difficult for participants to attend and to concentrate during sessions, ultimately reducing their work performance and ability during the treatment sessions. One participant expressed his opinion:

We are struggling with transport to the assessments. I struggle to come because I don't have money then I must borrow money from someone to get here. (Isaiah)

Another participant questioned where she might get exposure to get into work settings and gain access to real-life work experience after her head injury as there were no opportunities for her; she said:

Where must I get exposure for jobs? they all have reasons not to employ me like that manager at my sister's work. He just thought I'm dangerous or crazy and when I look at the stuff we do here and how slow I am, they won't accommodate me, for my slow speed, but if I had one arm missing they would have accommodated me. (Ruth)

### 10.2. Theme 2: The Model Helps Facilitate Work Integration (Enablers)

The above theme is indicative of the participants' positive experiences regarding the use of the MOOSE. Despite the limitations of the model, the advantages of the model outweighed the disadvantages, and holistically, participants reported an overall positive experience.

#### 10.2.1. Category 1: The Importance of Achieving Stage 1

The female participants were more prone to depressive symptoms and really battled in stage 1 in order to accept their new potential, as they always compared their current abilities to their abilities before the injury. One participant expressed her unhappiness with her new self after the injury:

I don't like the new normal. (Ruth)

I don't know. I just don't like the new norm… I think it's because it's too much…. (Ruth)

The above quotes emphasised how challenging it was to create and accept the new self after the injury. In conjunction with these statements, a simple observation during the process revealed how stressful it was for participants to accept their new abilities. An incident that provided evidence of the value in meeting the achievements in stage 1 was as follows:

Extract: *Throughout my experience, it has become evident that stage one is a very important stage in the model, if not the most important stage. During this stage the client has an opportunity to become aware of his or her new norms after the injury, aware of it and acceptance of it. It is the one stage where the therapist can either build the clients motivation to continue therapy or completely demolish it, before the other vocational rehabilitation can take place. The therapist has the opportunity in this stage to recreate a whole person again after the Injury, to accept their new work potential and create a healthy, realistic self – image of the clients and from this they can reach for jobs that are within their grasp and within their new working potential which will inevitably give them a new sense of living and being productive*. ([30.11.2013]—simple observation note)

#### 10.2.2. Category 2: Multiple Treatment Approaches

Another facilitator of the model was the multiple treatment approach, using both individual and group sessions in order to facilitate the progress of the participants. During stage 1, both approaches were utilized; during stage 2, more individual attention was given; and during stage 3, both approaches were again utilized. Participants described their experiences and highlighted what was beneficial to them:

The groups helped me realize I was not the only one with the problem, I am not alone and it even showed me that I am physically and mentally better off than some of the others, and I even got a chance to advise other people, which made me feel like a human again. (John)

#### 10.2.3. Category 3: The Importance of Occupational Engagement and the Advancement of Skills through Stage 3

This category represents the participants' experiences during stage 3, their perception of the work-simulated tasks in a controlled and uncontrolled (real-life) work situation. In the work area, the input or assistance from the therapist decreases and the participants now have to rely more and more on themselves during the treatment sessions. Participants emphasised what was important to them during this stage and why it was beneficial to them. One participant said:

Physical is better, because when you work you going to use physical, that is why I liked the hard work more, working there with the people. (Job)

#### 10.2.4. Category 4: A Holistic Experience

The participants gained satisfaction and meaning in engaging in the program; this theme emerged due to the positive feedback that was given to the therapist and highlighted their holistic experiences of the model and the program. The following are quotes highlighted whereby the participants placed value on their ability to remember instructions that before the intervention they used to forget. One participant said:

Yes, it helped me in the salon; It helped me to remember a lot because I used to forget. (Job)

### 10.3. Theme 3: Further Enhancements to Improve the Model

This theme represents the recommendations that the participants had with regard to further enhancements to the model and return to vocational rehabilitation programs.

#### 10.3.1. Category 1: Education on Holistic Health

This category represented the need for more education on overall health in the participants' lives. Some participants felt that they did not gain as much physical strength as they would have liked to receive and that they would have liked more education. One participant said:

I think you could have educated us more, not just our diagnosis but also how to live a healthy lifestyle, what to eat, the exercises that we got it was nice, I enjoyed that, but you can tell us how to keep our bodies fit and strong. (John)

#### 10.3.2. Category 2: An Increase in Social Support Groups

The participants reported the need for more social support groups; the quote below indicated how this specific participant had a need for more social support as he was not receiving any support in his area and coming to therapy was the only source of support he received.

He said:

I would like to have more groups because the others have the privilege of getting support in Khayelitsha, but me coming from Strand I only get the group here in the therapy, so groups are fun and I make friends, I get here what I don't get home. (Joshua)

#### 10.3.3. Category 3: Coping Skills and Contingency Plans

The above category represented the participants' need for coping skills and contingency plans while awaiting placement in a job. In Barriers, it will be discussed that participants often experienced despondency and a diminished sense of self-efficacy due to the lack of employment opportunities and stigma that still controls what kinds of employment, if any, TBI individuals obtain. Below is a quote that revealed the participant's need for more coping skills in waiting for a return to work opportunity and contingency plans. He said:

Now I am on probation at work, but they are all better than me, what if I am not good enough while I'm there, maybe I can't cope properly because the others are more capable than me. (Daniel)

Below, another participant expressed his frustration with possible job placements that did not get back to him after putting him through a rigorous interview, and he spoke how he still has no money, again showing that the model not necessarily guarantees a positive outcome. He said:

Now after this, she is disappointing us now because I had my hopes on the McDonalds job and now she (HR manager) is not even coming back to us, so what am I supposed to do at home, I don't have money, and things were not getting better at home so I am angry and I need to be active at home. (Isaiah)

## 11. Discussion

### 11.1. Barriers

The WHO [16] defines barriers to be factors that through their absence or presence in an individual's environment limit the individual optimal functioning and create disability. The term barrier signifies factors that negatively impact or hinder the participation of TBI survivors in the resumption of their worker role.

#### 11.1.1. The Transportation and Money Limitation

As discussed in the literature review and in the findings, most of the participants were unemployed and had been jobless for an extended period and some did not even have food or money to buy “pap” (maize meal) in order to concentrate during sessions. This added unnecessary stress on the participants and unnecessary strain on the therapist during sessions. For the study, a R60 fee was given to each participant for every session they attended; this amount was to cover transport and food. Largent and Lynch [17] argue that the provision of payment for participation in research-related activity is acceptable as long as the research participants are not coerced to participate in the study.

#### 11.1.2. Individual Choice of Activity

Activities that were unfamiliar and lacked meaning for the participants tended to be administrative-related activities. This was mostly due to the participants' lack of exposure to clerical-related tasks as most of the participants were employed in a “blue-collar” type of occupations prior to their injury. In this study, the participants indicated that engagement in the work-related activities during the program increased their self-worth through being independent and competent in gaining skills. Chamberlain et al. [18] described work as integral to how people define themselves. The consequences of unemployment affect an individual from both psychosocial and financial perspectives. It could therefore be argued that the inability of people with traumatic brain injury to find and maintain employment is more severely affected than the general population [11]. Therefore, the role of returning to work and regaining to their roles as a productive member of society can never be underestimated.

#### 11.1.3. The Lack of Funding for Vocational Rehabilitation Programs in SA

The findings of the current study indicated that a limitation of the program's success was a lack of resources in terms of funding, and this lack of funding contributed to the shortage of vocational rehabilitation services being offered in South Africa as a whole. Most of the budget for the health sector goes into primary health care for the purpose of preventing disease and promoting health. This lack of funding is significantly impacting the vocational rehabilitation services in SA [19] and is hindering vocational rehabilitation services that could assist people with TBIs to return to work successfully. Results of this study indicated that a lack of funding was caused by a bigger problem of a lack of legislation or the lack of practical implementation of policies to facilitate the reintegration of people with disabilities (PWD) in the workplace [20].

#### 11.1.4. The Unemployment Crisis in South Africa

The findings of the study showed that despite legislation, PWD were still not being employed. Not only due to stigma and ignorance on the employers' part but also due to the fact that the unemployment rate in SA is extremely high and they are limited to minimal employment opportunities available for the able-bodied and disabled SA citizens alike. The findings from the study revealed that most participants were the recipients of disability grants despite the fact that tests showed that they did not meet the criteria for a disability grant cognitively and physically yet were receiving this financial assistance due to their impoverished social circumstances. A limitation to the MOOSE was that despite all efforts of going through the model, it did not guarantee a positive outcome (not all of the participants were successfully placed in working environments) which leads to demotivation and a reduction in self-efficacy.

### 11.2. Facilitators

The facilitators include importance of stage 1—goal setting and getting introspection—and importance of occupational engagement and enhancing work skills.

In the current study, stage one proved to be a very important stage as the participants described that stage one allowed them an opportunity to reflect on their functional challenges and allowed them to set goals in terms of their work goals. Supportive employment models enable individuals with disability to develop work skills within a real work setting [21]. However, the MOOSE differs from traditional supportive employment models in that it is a client-centered model that enables the individual with brain injury to introspect using the Gibbs reflective cycle and play an active role in choosing as well as directing work skills training. Similar to supportive employment models described by Kirsh et al. [22] on stage four of the MOOSE, the individual with the brain injury continues to utilize the support from the case manager or occupational therapist (job coach). There is however a strong indication in the supportive employment literature that ongoing support in the workplace could be viewed negatively by coworkers and supervisors may perceive this type of support as different to traditional workplace training offered by employers [23].

### 11.3. Enhancements to the Model of Occupational Self-Efficacy

Despite the findings of the study suggesting that the MOOSE should have a greater emphasis on having sessions that focus on healthy diet, utilizing support groups, and enhancing coping skills, the findings also highlight a concern specifically related to how the model can assist an individual who experiences poverty and unemployment especially when the individual struggles to complete vocational rehabilitation and improve their work skills. It could be argued that more emphasis needs to be placed on having work placement agencies and skills training authorities such as the Sector Education Training Authority (SETA) be involved in providing training opportunities particularly in stage 3 and stage 4 of the model. The SETA programs are training programs developed by the South African government that enable an individual to train for a specific job while receiving a stipend or financial allowance while they are completing their training [24].

The researcher is of the opinion that with the provision of work opportunities from the government and or companies in the private sector for the people with disability (such as individuals who sustained a traumatic brain injury), the effects of poverty and unemployment may be reduced. The partnering with both companies in both the public and private sectors so that individuals may be allowed a financial stipend to support themselves especially when they are completing vocational rehabilitation programs may contribute to an enhanced return to work rate.

## 12. Limitations of the Study

One major limitation that was identified in this study was the inability to generalise the findings of this study to the larger population due to the inherent nature of qualitative research and the limited number of study participants. Another limitation was the fact that mainly male participants participated in the study. Due to the nature of brain injuries more males tends to be affected with brain injuries than females.

## 13. Conclusion

This study explored the experiences and perceptions of individuals who sustained a brain injury regarding returning to work after participating in a vocational rehabilitation program. The vocational rehabilitation program used the stages of the MOOSE in returning individuals with brain injury to work. Some of the barriers identified in the study included miscommunication and misperception, stage two is too frustrating, and general lack of resources and employment opportunities. Some of the facilitators included utilizing multiple treatment approaches, importance of occupational engagement and the advancement of skills through stage 3, and a holistic experience. The recommendations to improve the model included education on holistic health, an increase in social support groups, and the inclusion of coping skills and contingency plans. The findings of this study indicated that the MOOSE is a useful model to use in retraining work skills to an individual with brain injuries. The participants in this study could maintain employment in the open labour market for a period of at least 12 months, and it improved their ability to accept their brain injury as well as adapt to their worker roles. A limitation to the MOOSE was that despite all efforts of going through the model, it did not guarantee a positive outcome (not all of the participants were successfully placed in working environments) which leads to demotivation and a reduction in self-efficacy making it counterproductive. As MOOSE works on improving self-efficacy as a key factor for improving the individual with TBI's work ability, the opposite can also occur, i.e., an individual's self-efficacy beliefs can also deteriorate if their functional ability does not improve. It could be argued that developing an unrealistic sense of self-confidence in one's work skills could result in an individual developing an unrealistic view or belief in their ability to do jobs that they are not capable of doing due to their educational level or functional ability.

### 13.1. Implications for Practice

- The Model of Occupational Self-Efficacy is a useful model to use in retraining work skills
- Social support groups and coping skills are important in rehabilitation programs
- Improving self-efficacy beliefs is a key factor for improving an individual's work ability

## Figures and Tables

**Table 1:** Demographics of the participants (reproduced from Shaheed Soeker, “The Use of the Model of Occupational Self Efficacy in Improving the Cognitive Functioning of Individuals with Brain Injury: A Pre- and Post-Intervention Study,” *Work*, Vol. 58, 2017, pp. 63-72 (under the Creative Commons Attribution License/public domain)).

Names	Age	Gender	Education	Marital status	Diagnosis	Employment prior to injury	Tx prior to rehab
Peter	28	Male	Grade 10	Single	Mild frontal	Security guard	None
Matthew	34	Male	Grade 7	Divorced	Moderate frontal and parietal lobe	Security guard	Hand Tx
Esther	33	Female	Tertiary	Single	Moderate frontal	Jewelry designer	None
Isaiah	34	Male	Grade 11	Single	Mild frontal and temporal	Petrol attendant	Support group
Job	30	Male	Grade 10	Single	Mild frontal	Hair salon	Support group
Joshua	28	Male	Grade 10	Single	Moderate frontal & parietal	General worker	Hand Tx
Ruth	33	Female	Tertiary	Single	Mild frontal & occipital	Bank teller	None
John	20	Male	Grade 12	Single	Mild parietal	General worker	Support group
Luke	36	Male	Grade 10	Single	Mild frontal & parietal	General worker	Support group
Daniel	21	Male	Grade 11	Single	Mild frontal	General worker	Support group

## Data Availability

The interview data used to support the findings of this study are restricted by the Human Research Ethics Committee, University of the Western Cape, in order to protect participant privacy. Data are available from Mogammad Shaheed Soeker (msoeker@uwc.ac.za) for researchers who meet the criteria for access to confidential data.
